# An overview of the mechanisms and potential roles of extracellular vesicles in septic shock

**DOI:** 10.3389/fimmu.2023.1324253

**Published:** 2024-01-26

**Authors:** Meiling Cao, Mingyue Shi, Boru Zhou, Hongkun Jiang

**Affiliations:** ^1^ Department of Neonatology, The First Hospital of China Medical University, Shenyang, Liaoning, China; ^2^ Department of Pediatrics, The First Hospital of China Medical University, Shenyang, Liaoning, China

**Keywords:** diagnosis, extracellular vesicles (EVs), nanoscale vesicles, pathogenesis, septic shock, treatment

## Abstract

Septic shock, a subset of sepsis, is a fatal condition associated with high morbidity and mortality. However, the pathophysiology of septic shock is not fully understood. Moreover, the diagnostic markers employed for identifying septic shock lack optimal sensitivity and specificity. Current treatment protocols for septic shock have not been effective in lowering the mortality rate of patients. Most cells exhibit the capability to release extracellular vesicles (EVs), nanoscale vesicles that play a vital role in intercellular communication. In recent years, researchers have investigated the potential role of EVs in the pathogenesis, diagnosis, and treatment of different diseases, such as oncological, neurological, and cardiovascular diseases, as well as diabetes and septic shock. In this article, we present an overview of the inhibitory and facilitative roles that EVs play in the process of septic shock, the potential role of EVs in the diagnosis of septic shock, and the potential therapeutic applications of both native and engineered EVs in the management of septic shock.

## Introduction

Every year, millions of people worldwide endure major medical complications related to sepsis and septic shock, resulting in fatalities in one-third to one-sixth of patients who are affected (1). As a dysfunctional systemic inflammatory disease, sepsis can lead to organ damage, which can pose a serious threat to the lives of patients (2). Sepsis is now identified as a life-threatening organ dysfunction caused by a disordered host response to the infection (3). Sepsis, severe sepsis, and septic shock are systemic inflammatory responses to an increasingly severe infection. In the most serious cases, sepsis leads to multiple organ dysfunction and can cause severe immune dysfunction and catabolism, leaving the patient in a chronic, critical condition (3). Septic shock is a subset of sepsis that has a greater risk of death than sepsis alone due to its particularly severe circulatory, cellular, and metabolic abnormalities (4).

Clinically, patients with septic shock need vasopressors to maintain a mean arterial pressure of 65 mmHg or higher, and their serum lactate levels are higher than 2 mmol/L (>18 mg/dL) in the absence of hypovolemia (4). Procalcitonin (PCT) and C-reactive protein (CRP) are commonly used as markers to diagnose sepsis and septic shock; however, both of these, as well as the neutrophil marker CD64, currently have limited specificity and sensitivity as sepsis biomarkers (5).

Extracellular vesicles (EVs), a collection of heterogeneous cell-derived membrane structures, include exosomes shed from the plasma membrane and vesicles originating from the endosomal system (6). The nomenclature for EVs varies based on different classification criteria: a) size; b) density; c) biochemical composition; and d) descriptions of conditions or cells of origin. In this review, we have used operational terms relating to EVs (7). Microvesicles (MVs) typically exhibit larger dimensions, ranging approximately between 100 and 1000 nm. These vesicles, arising from budding or outward protrusions of the plasma membrane, are also included within the broader classification of EVs (6). In addition, new classes of EVs, such as migrasomes, have been identified (8).

EVs vary widely in composition. Some of them are rich in ubiquitinated proteins, heat shock proteins, and membrane proteins such as integrins, MHC I, MHC II, and the four-transmembrane proteins CD63, CD9, and CD81 (9). These can be transferred to target cells via intercellular communication and perform vital functions such as antigen presentation, immune monitoring, and modulation of inflammatory responses (10). In addition, EVs can transport lipids or nucleic acids, enabling the regulation of receptor cells and controlling signaling pathways (11, 12).

EVs from different types of cells have been proven to play distinct roles in sepsis. EVs orchestrate intercellular communication, thereby mediating several pathophysiological processes (9). In patients with septic shock, EVs represent a novel mechanism of intercellular communication wherein they deliver miRNA and mRNA associated with pathogenic pathways, including those related to inflammatory responses, oxidative stress, and cell cycle regulation (12). In the context of sepsis, EVs exert a dual role: to promote or suppress the development of sepsis by regulating the immune response and endothelial function (13). A growing body of data suggests that microRNA, non-coding RNA, and EVs can be considered novel markers to diagnose sepsis (14–17). Furthermore, based on a thorough review of existing literature on the pathophysiology of septic shock, we opine that EVs have substantial potential for managing septic shock (18, 19).

In summary, EVs play a vital part in sepsis and septic shock. Hence, in this review, we have summarized current findings regarding the role of circulating EVs in sepsis and septic shock and have outlined the potential of EVs as a diagnostic marker for sepsis as well as their role in treating sepsis and septic shock.

## The role of EVs in the pathogenic pathways in septic shock

The interaction between EVs and recipient cells relies on the specific surface receptors on the membrane of EVs and their corresponding ligands on the surface of target cells. EVs can be absorbed by target cells through receptor-ligand binding, facilitating the transfer of their contents to the cytoplasm of target cells. The substances carried by EVs can affect the function of target cells by stimulating signaling pathways or providing nutritional support (6). Intercellular exchange facilitated by EVs is based on proteins, the transport of ribonucleic acids (mRNA, miRNA, and noncoding RNA), and DNA sequences, the most relevant of which are induced by microRNAs (miRNAs). A comprehensive analysis of serum EVs from patients with sepsis revealed significant alterations in 65 exosomal miRNAs and that the pathways of miRNA enrichment in patients with sepsis are mainly related to the inflammatory response (12). Consequently, we have summarized the roles of EVs in the pathogenic pathways of sepsis and septic shock.

## EVs are involved in relieving the progression of septic shock

**Figure 1:**
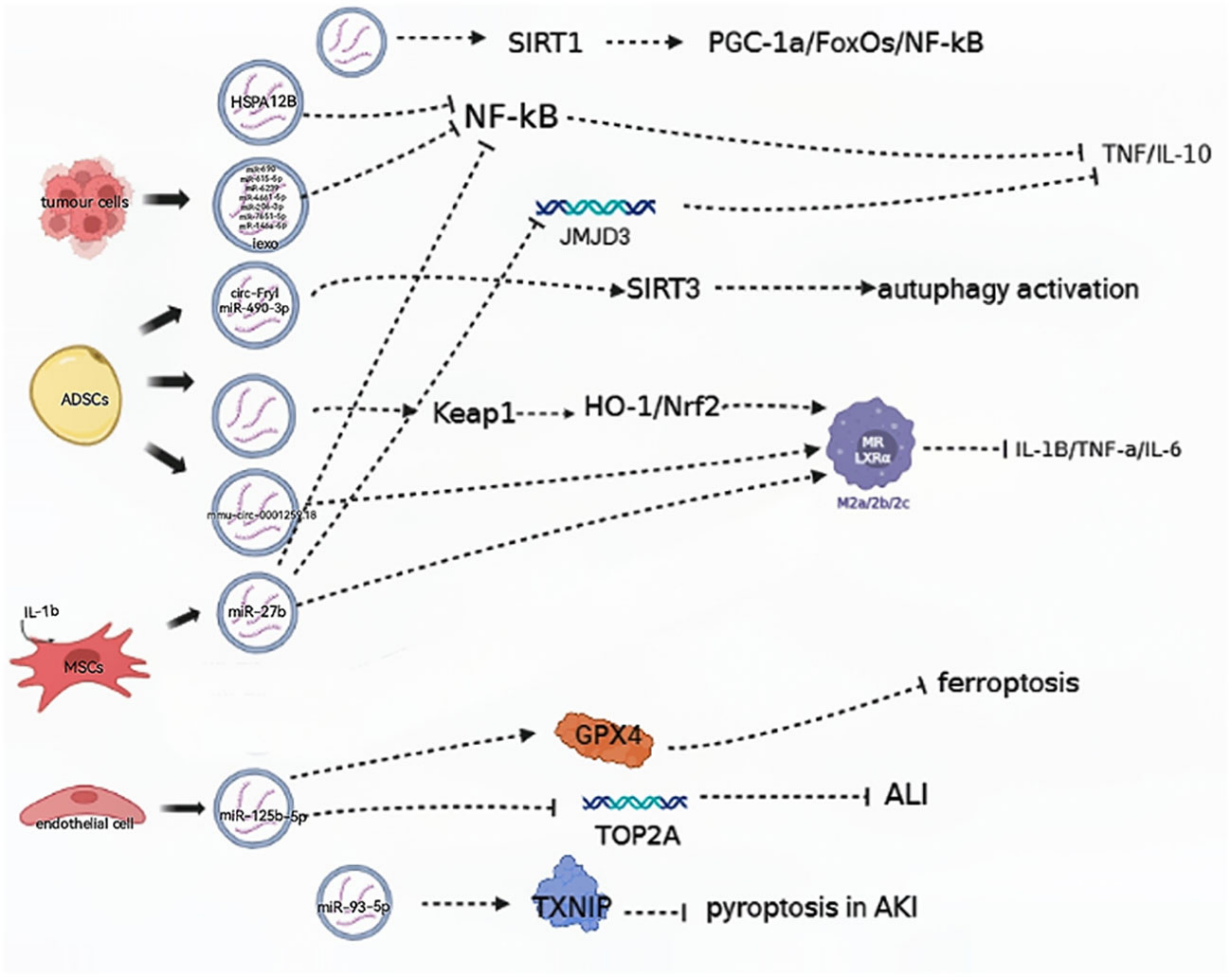
EVs are involved in aggravating the progression of septic shock. (→): upregulate; (—): downregulate; ADSCs, adipose-derived stem cells; MSCs, mesenchymal stem cells; HO-1, heme oxygenase-1; Nrf2, nuclear factor erythroid 2-related factor 2; NF-κB, nuclear factor kappa-B; GPX4, glutathione peroxidase 4; TXNIP, thioredoxin-interacting protein.

EVs deliver a variety of nucleic acids, proteins, cytokines, and other substances to regulate immunity and endothelial function, alleviate multiple organ failure, and, subsequently, relieve sepsis and septic shock. (**Figure 1**) During sepsis, macrophages can absorb EVs containing endothelial HSPA12B. In a mouse model study, it was found that HSPA12B carried by EVs reduces the production of TNF-α and IL-1β by downregulating the activation of NF-κB, thereby reducing the LPS-stimulated inflammatory response (20). In a study on mice subjected to cecal ligation and puncture (CLP mice), mice in the experimental group were injected with adipose-derived stem cell (ADSCs) EVs. It was found that EVs were involved in upregulating heme oxygenase-1 (HO-1) and nuclear factor erythroid 2-related factor 2 (Nrf2) expression by downregulating Kelch-like ECH-associated protein 1 (Keap1). They also had a role in polarizing macrophages to an anti-inflammatory phenotype, alleviating lipopolysaccharide (LPS)-induced reactive oxygen species (ROS) accumulation, expression of inflammatory factors such as IL-1β, TNF-α, and IL-6, relieving LPS-induced inflammation, and improving the inflammatory response and damage of multiple organs in sepsis (21).

Gao et al. found that EVs isolated from mice with sepsis contained all 18 cytokines, chemokines, and growth factors, offering the first evidence that EVs from mice with sepsis have immunoreactive properties. Additionally, these EVs promote T cell proliferation and enhance T lymphocyte migration through the actions of IL-2 and IL-4 cytokines, thereby effectively enhancing Th1/Th2 differentiation (10). Furthermore, the nuclear translocation of NF-κB activates the transcription of several inflammatory cytokines, including TNF-α and IL-6, and eventually triggers cytokine storms during sepsis (22).

In a mouse model, tumor cells demonstrated the ability to release induced EVs (iExo) subsequent to treatment with LPS, and this suppressed NF-κB expression and nuclear translocation. Notably, seven miRNA species in iExo were found to be specifically upregulated to alleviate sepsis by regulating immunity and endothelial function (23). Additionally, mesenchymal stem cells (MSCs) are capable of secreting EVs containing specific miRNAs, and researchers found that in CLP mice, EV miR-27b derived from MSCs was effective in hindering the recruitment of JMJD3 and NF-κB in the promoter region of proinflammatory genes, thereby reducing the expression of these genes and inhibiting sepsis (24).

Exosomes derived from ECs that overexpressed miR-125b-5 were found to prevent acute lung injury (ALI) induced by sepsis by inhibiting TOP2A and alleviate inflammation-induced iron death by upregulating GPX4. Meanwhile, ADSC-derived Evs increased the expression of Nrf2 and nuclear translocation and reduced Keap1 expression in CLP mice (25). ADSC-derived Evs were found to promote autophagy activation by delivering circ-Fryl and modulating the miR-490-3p/SIRT3 pathway. This autophagy activation could then suppress sepsis-induced lung injury by reducing apoptosis and inflammatory factor expression (26).

Studies have suggested Evs may ameliorate acute kidney injury (AKI) induced by CLP via the sirtuin-1 (SIRT1) signal pathway. SIRT1, a member of the sirtuin family of proteins, is known to regulate cell survival and apoptosis through a variety of mechanisms. Recently, researchers identified PGC-1α, FoxOs, and NF-κB as key targets of SIRT1 in regulating sepsis-induced inflammation (27). Furthermore, in CLP mice, ADSC-derived EVs, especially those pretreated with hypoxic (HExo), were found to deliver mmu_circ_0001295. This ameliorated increases in plasma levels of chemokines and cytokines as well as prolonged renal injury induced by sepsis (28).

EVs can be involved in slowing the pathological processes of sepsis-induced AKI by regulating proteins. Chen et al. found that EV miR-93-5p plays a role in inhibiting pyroptosis during the advancement of AKI by regulating thioredoxin-interacting protein (TXNIP). They suggest that EV miR-93-5p contributes to varying effects on the levels of pyroptosis in renal tubular epithelial cells, depending on the phenotypes of macrophages, and these findings also highlight that the same EVs can lead to varying outcomes when acting on different types of macrophages (29).

## EVs are involved in aggravating the progression of septic shock

**Figure 2:**
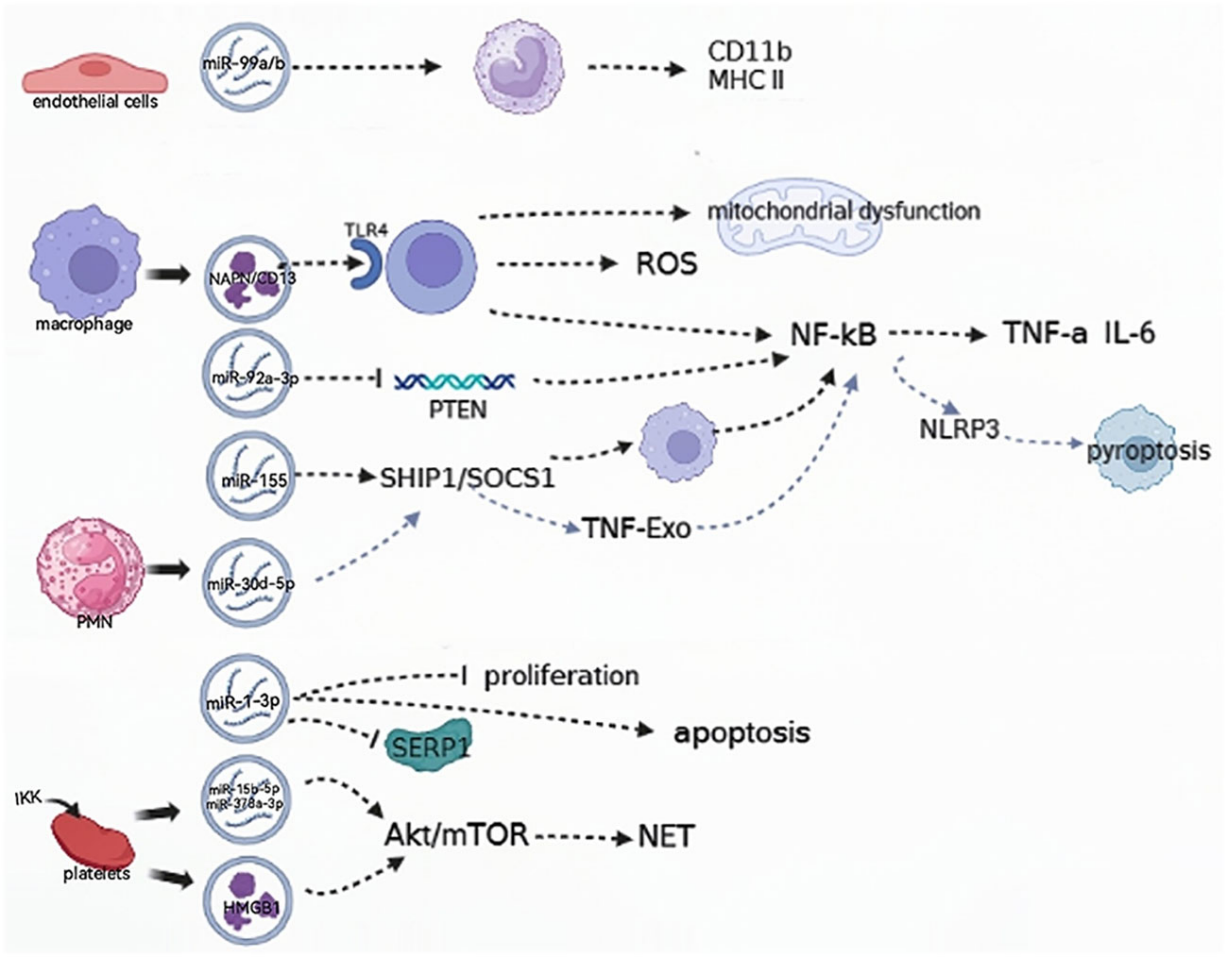
EVs are involved in relieving the progression of the septic shock. (→): upregulate; (—): downregulate; ROS: reactive oxygen species; SHIP1, SH2-containing inositol phosphatase 1; SOCS, suppressor of cytokine signaling1; NLRP3, NOD-like receptor thermal protein domain associated protein 3; TNF-Exo, EVs released by TNF-α-stimulated PMNs; PMN, polymorphonuclear neutrophil; SERP1, endoplasmic reticulin protein 1; IKK, IκB kinase; HMGB 1, high-mobility histone 1; mTOR, mechanistic targets of rapamycin; NET, neutrophil extracellular trap.

EVs play a key role in modulating immunity, regulating the endothelium, and mediating multiple organ damage to promote the development of septic shock (**Figure 2**). EVs from *Staphylococcus aureus*-infected endothelial cells were found to facilitate the expression of CD11b and MHC II in monocytes and lead to the dysregulation of cytokine production. When infected by *Staphylococcus aureus*, endothelial cells release EVs, which contain the key miRNAs miR99a and miR99b. When these EVs are absorbed by immune cells, they target the mechanistic targets of rapamycin (mTOR), subsequently triggering an uncontrolled release of proinflammatory cytokines. Collectively, the dysregulation of miR-99a/b expression in endothelial EVs drives a proinflammatory phenotype in monocytes (30, 31).

Neutrophil extracellular traps (NETs) are intricate webs of DNA and proteins that are released by neutrophils to trap and eliminate bacteria. EVs containing high-mobility histone 1 (HMGB 1) and/or miR-15b-5p and miR-378a-3p can induce the formation of NETs via the Akt/mTOR autophagy pathway. This promotes subsequent organ damage and sepsis progression, and the IκB kinase (IKK) controls platelet-derived EVs secretion in CLP mice (32). In patients with septic shock, the functional characterization of serum exosomal differential mRNA includes humoral immune responses mediated by antimicrobial peptides, ubiquitination of target proteins by E3 ubiquitin ligase, and secretion of cytokines. The significant enrichment of these functional terms indicates the complexity and specificity of the immune response and inflammatory response mechanisms in patients with septic shock (16).

EVs play a crucial role in sepsis by inducing inflammation through the activation of cells and their contents (33). ALI induced by sepsis is a severe complication of sepsis and a leading cause of mortality. The human plasma EV aminopeptidase NAPN/CD13 from macrophages binds to TLR4, inducing ROS generation, mitochondrial dysfunction, and NF-κB activation, which regulate the necroptosis of lung epithelial cells (34). The miR-155 protein derived from the serum EVs of ALI mice was found to promote the proliferation and inflammatory responses of macrophages by targeting SHIP1 and SOCS1. This process stimulates the activation of NF-κB and triggers the production of TNF-α and IL-6, respectively (14).

Pulmonary inflammation can be induced by EVs obtained from alveolar epithelial cells (AECs) that are treated with LPS. EVs transport miR-92a-3p, which facilitates communication between AECs and alveolar macrophages (AMs) and enhances the activation of macrophages. This occurs by suppressing PTEN expression, and regulating the activation of the NF-κB signaling pathway, and finally resulting in the development of ALI (30, 35). In addition, it was found in another study that the decreased expression of sepsis plasma-derived miR-1-3p stress-associated endoplasmic reticulin protein 1 (SERP 1) was associated with the upregulation of miR-1-3p. This hindered the growth of cells, accelerated cell death, and caused contraction of the cytoskeleton, resulting in increased permeability of monolayer endothelial cells and damage to the membrane. Consequently, these changes lead to endothelial cell dysfunction and vascular barrier dysfunction related to the progression of ALI (36).

PM (polymorphonuclear neutrophil)-EV regulates the function of macrophages by carrying miR-30d-5p molecules. Specifically, miR-30d-5p in PMN-EV can target the SOCS-1 and SIRT-1 molecules in macrophages, thereby mediating the function of TNF-Exo (EVs released by TNF-α-stimulated PMNs). In this process, TNF-Exo induces the expression of the NLRP3 inflammasome via the activation of the NF-κB signaling pathway, thereby promoting the inflammatory response and the development of ALI (37). Xiang et al. showed that macrophage-derived EVs can cause glomerular endothelial cell dysfunction. By regulating acid globulase (ASM), these EVs are released extracellularly during LPS-induced endotoxemia, triggering cell-cell communication and leading to glomerular endothelial cell dysfunction in mice (38).

## Role of EVs in the diagnosis of septic shock

More than 250 biomarkers for sepsis have been evaluated in clinical and experimental investigations (39). Studies have evaluated the diagnostic and prognostic potential of several novel biomarkers in emergency situations (39). Emerging biomarkers of sepsis include presepsin, sTREM-1, proadrenomedullin, transcriptomics, proteomics, and metabolomics (40). However, in the present review, we have summarized current findings on the possible relevance of circulating EVs in the diagnosis of sepsis and septic shock (**Table 1**).

**Table 1:** Roles of EV in the diagnosis of septic shock.

Cargo	Models	Note	Reference
DNMT mRNAs DNMT3A DNMT3B	humans	EVs from septic shock patients carried more total DNMT mRNAs and more DNMT3A+DNMT3B mRNAs than control subjects or sepsis EVs.	(41)
Protein-1 (PRDX 1)	humans	PRDX 1 were present only in patients with sepsis	(16)
SPTLC3	humans	SPTLC3 was negatively correlated with the progression of sepsis	(42)
Particulate actin	humans(plasma)	the presence of particulate actin in the bloodstream is indicative of cell damage caused by sepsis	(43)
miR-92a-3p	mice	the activation of alveolar macrophages is promoted by exosomes derived from alveolar epithelial cells, through the mediation of miR-92a-3p.	(35)
miR-30d-5p	mice	Exosomal miR-30d-5p from PMNs induced M1 macrophage polarization and prime macrophage pyroptosis.	(5)
APN/CD13	mice	exosomes secreted by LPS-stimulated macrophages exhibit an exceptionally elevated level of APN/CD13 expression.	(34)
miR-155	mice	there was a high presence of exosomes loaded with microRNAs (miRNAs), such as miR-155, in the peripheral blood of mice with ALI.	(14)
miR-150-5p	NA	miR-150-5p, in combination with NT-pro BNP, LVEF, and SOFA score, can be used as an independent predictor for septic cardiomyopathy.	(15)
CD105+Evs CD31+ EVs	humans	patients with meningococcal septic shock showed higher procoagulant activity in EVs compared to non-shock patients.	(44, 45)
miR‐125b‐5p, miR‐26b‐5p,miR-27b-3p	humans mice	The miR-27b-3p was upregulated in both exosomes and blood cells from septic patients.	(46)
miR-27b	mice	Exosomes derived from MSCs show high expression of miR-27b in septic shock.	(24)
miR-15a, miR-16	humans(plasma)	serum miR-15a and miR-16 are capable of differentiating between individuals with sepsis/SIRS and those without any medical condition.	(47)
eCIRP	mice	after treatment with LPS, the concentration of eCIRP within the exosomes showed a significant increase.	(48)
Y-RNA	humans	the ratios of Y-RNA isoform in plasma EVs were strongly correlated with the changes in circulating neutrophil and monocyte numbers induced by inflammation.	(17)

NA, not appliable.

The elevation in total plasma EV count and exosomal EV-DNMT mRNA load may serve as a new reference for the diagnosis of sepsis and septic shock. The levels of EV-DNMT1 and EV-DNMT3A+DNMT3B and the severity of sepsis and septic shock were found to be strongly correlated. It was observed that the EVs from patients in septic shock contained a higher quantity of total DNMT mRNAs, as well as DNMT3A+DNMT3B mRNAs, when compared to EVs from control subjects or individuals with sepsis. Additionally, the severity of sepsis was also associated with the total number of plasma EVs. Therefore, the load capacity of EV-DNMT mRNAs, in conjunction with the total number of plasma EVs, could serve as a novel diagnostic approach for identifying the time when patients with septic shock need intensive care (41). This method could also provide opportunities for more targeted interventions, such as regulating the release of EVs or controlling specific DNMT activity, in order to improve the effectiveness of standard therapy or other treatment strategies. In another study, the severity of organ failure and the mortality rate in critically ill patients with sepsis were found to be linked to higher levels of plasma EVs (49).

Isolating EVs for proteomic analysis shows promise as a potential method for the diagnosis of sepsis and assessing prognosis. The progression of sepsis involves the dysregulation of 99 proteins, and some of these, such as peroxide reduction protein-1 (PRDX 1), were found to be present only in patients with sepsis. This finding suggests that these proteins can be potential diagnostic marker candidates, meriting further validation (16).

Xu et al. optimized an EV preparation method involving one-step ultracentrifugation of serum EVs with an Optiprep™ cushion and performed mass spectrometry analysis using Q-Exactive plus. This enabled researchers to deeply analyze exosomal proteomics in human blood on the scale of clinical studies. Additionally, they found that serine palmitoyltransferase 3(SPTLC3) was negatively correlated with patterns of disease progression in sepsis (42). SPTLC3 can be a potential classifier for monitoring the clinical progression of sepsis in the future. In addition, it was shown in another study that actin levels were increased in serum EVs, while the levels of the actin-binding protein gelsolin (GSN) were decreased (16). However, previous research has established that the presence of particulate actin in the bloodstream is indicative of sepsis-induced cell damage, and low levels of plasma GSN have been identified as a marker for a negative prognosis in sepsis (43).

In the pathophysiology of sepsis and septic shock, multiple EV-borne miRNAs are up-/down-regulated. Sepsis-induced ALI involves the mediation of miR-92a-3p by EVs derived from alveolar epithelial cells to promote the activation of alveolar macrophages (35). In instances of sepsis-induced ALI, the activation of NF-κB signaling leads to M1 macrophage polarization and triggers macrophage pyroptosis, thus contributing to the development of sepsis-related ALI. This effect is mediated by the miR-30d-5p molecules in EVs derived from PMNs (37). Furthermore, miR-125b-5p present in EVs from ADSCs can alleviate inflammation-induced ferroptosis in pulmonary microvascular endothelial cells (PMVECs) during sepsis-induced ALI by regulating the expression of Keap1/Nrf2/GPX4. Therefore, the presence of miR-125b-5p helps in the recovery from ALI during sepsis (25).

EVs secreted by LPS-stimulated macrophages were found to exhibit an exceptionally elevated level of APN/CD13 expression, and APN/CD13 present in EVs was found to be capable of inducing necroptosis in epithelial cells, contingent on the presence of APN/CD13 (34). Another investigation found that there was a high presence of EVs loaded with miRNAs, such as miR-155, in the peripheral blood of mice with ALI (14). These studies suggest that miR-92a-3p in AEC EVs, exosomal miR-30d-5p from PMN, exosomal miR-125b-5p derived from ECs, exosomal APN/CD13, and miR-155 mediated by peripheral circulating EVs represent novel and potential diagnostic biomarkers for sepsis-induced AKI that can contribute to the diagnosis of sepsis and septic shock.

Further research is needed to determine the potential of using miR-150-5p in combination with the NT-pro BNP, LVEF, and SOFA score as an independent predictor for the progress of septic cardiomyopathy. However, findings deduced from a multivariate logistic regression analysis in a study suggested that miRNAs found in EVs derived from neutrophils could play a vital role in the severity of septic disease progressing towards cardiomyopathy. Therefore, while miR-150-5p holds potential to predict the severity of sepsis, additional investigation in this aspect is necessary (15). In another study on sepsis-associated AKI, macrophages were found to infiltrate more, and there was an increased secretion of EVs in the glomeruli among samples in the LPS-induced AKI group, resulting in dysfunction of glomerular endothelial cells (38). A study found higher procoagulant activity in EVs in patients with meningococcal septic shock compared to the non-shock patients, and the role of CD105+ EVs and CD31+ EVs as potential marker candidates requires further validation (44, 45).

The use of EV-derived miRNAs provides valuable insights into the diagnosis of sepsis and the prediction of survival, and they are also potential targets for developing new biomarkers for sepsis. MiR-199b-5p from blood cellular elements has been identified as a possible early indicator in this regard. EVs uniquely regulate miR-125b-5p, while serum specifically regulates miR-26b-5p. However, miR-27b-3p is present in three components, namely, serum, EVs, and cells. In septic patients, both EVs and blood cells were found to show an upregulation of miR-27b-3p (46).

Increased levels of exosomal hsa_circRNA_104484 and hsa_circRNA_104670 were found in sepsis serum EVs, suggesting their potential diagnostic value in detecting sepsis. MSCs-EVs overexpress miR-27b in septic shock. MiR-27b was found to specifically target Jumonji D3 (JMJD3) and reduce the expression of pro-inflammatory genes by inhibiting the recruitment of JMJD3 and NF-κB in the gene promoter region (24). Another study demonstrated that serum miR-15a and miR-16 are capable of differentiating between individuals with SIRS and those without any medical condition. In particular, miR-15a displays the potential to distinguish sepsis from SIRS, indicating its role as a marker for this differentiation (47).

EVs have the capability to release eCIRP and then trigger the production of cytokines and the migration of neutrophils. A study found that the concentration of eCIRP within EVs showed a significant increase after treatment with LPS (48). In addition, EV-associated Y-RNA has a potential role as a biomarker in immune-related diseases. In a human endotoxemia model, the ratios of the cell-type-specific Y-RNA isoform in plasma EVs were changed and strongly correlated with the number of circulating neutrophils and monocytes induced by inflammation (17).

For a compound to be considered a potential biomarker, it needs to be sufficiently stable so that it can be consistently detected in a hospital setting. The development of sensitive tests that can measure the levels of cellular and/or circulating compounds and metabolites has enabled the identification of distinctive “signatures” of possible diagnostic biomarkers in almost any biological sample, including blood (50) and urine (51). As diagnostic markers for sepsis and septic shock, EVs play important roles in the pathological process. Detecting EVs does not impair the sensitivity of pathogens in patients with sepsis and septic shock; however, it does not identify the infected pathogen, either. The sensitivity, specificity, and feasibility of EVs as diagnostic markers for sepsis and septic shock (that is, using a process that is easy to operate, offers accurate interpretation of test results, is not very complicated in terms of technical expertise requirements, and is cost-effective) need further investigation.

## EVs as potential therapeutic targets related to septic shock

In the past few years, researchers have explored the potential of EVs in disease treatment. There have been promising advances in the treatment of diseases with EV therapy. After comprehensively reviewing the available pathophysiological studies of septic shock, we conclude that EVs have great potential in the treatment of septic shock, even though its pathophysiology is not fully known (18, 19). Therapeutic EVs are commonly classified as native EVs and engineered EVs, both of which are used in clinical trials for the treatment of septic shock.

## Native EVs for the treatment of septic shock

The main native EVs currently used for *in vivo* treatment are mesenchymal stem cell (MSC)-derived EVs, dendritic cell (DC)-derived EVs, and plasma-derived EVs, which may be beneficial in treating septic shock. Boisramé et al. extracted EVs from the blood of septic shock rats treated with activated protein C (aPC) using multiple centrifugations. When injected into rats in septic shock, these EVs limited pro-inflammatory pathways and hemodynamic dysfunction and attenuated the decrease in mean arterial pressure induced by septic shock. Interestingly, the mean arterial pressure in rats with septic shock treated with aPC was not statistically different from that of rats in septic shock, as aPC reduced the amount of norepinephrine required to achieve the target mean arterial pressure (MAP), providing a new idea for isolating native EVs for therapeutic use (52).

## Engineered EVs for the treatment of septic shock

Various cargos can be harnessed to treat diseases through the engineering of EVs, including loading proteins, functional RNA, pharmaceutical compounds, surface-modified proteins, and surface-modified peptides onto EVs. Choi et al. loaded IκBα, a super inhibitor that inhibits the NF-κB pathway, into engineered EVs and found that injection of such EVs improved survival and reduced inflammation and acute organ damage in mice with sepsis (53). Extending this line of thinking, the super-repressor, IκBα, may have a similar effect on septic shock mice because inhibition of the NF-κB pathway was also found to improve survival in septic shock mice and decrease inflammation and acute organ damage (54). Ding et al. used macrophage-derived EVs loaded with siRNA that targeted the chemokine receptor CCR2, a mediator of inflammatory Mo/Mps chemotaxis. Targeting the spleen, these engineered EVs were effective in inhibiting inflammatory Mo/Mps chemotaxis in mice and could inhibit splenic Ly6C^high^ inflammatory monocyte mobilization and alleviate septic shock in mice (55).

The above two studies illustrate the potential of engineered EVs for treating septic shock. In the following section, we have summarized some cargos loaded into engineered EVs that can contribute to the treatment of septic shock.

## The role of cargos loaded into engineered EVs in inhibiting inflammation in septic shock

Sepsis and septic shock are fundamentally inflammatory diseases. Current targeted therapies for inflammation in septic shock have the following three main strategies: 1. inhibition of complement and cell surface receptors that recognize microbial products of infectious origin and endogenous danger signals, such as nod-like receptors, toll-like receptors, and rig-like receptors; 2. inhibition of key signaling pathways such as the MAPK pathway, the JAK-STAT pathway, and the NF-κB pathway; and 3. inhibition of key inflammatory cytokines such as IFN-γ, IL-6, IL-8, CCL2, and CCL3 (56).

PDK2-IN is a potent, competitive and selective inhibitor of PDK2. Li et al. found that suppressing PDK2 with this inhibitor lowered TLR4, which then inhibited the downstream JNK/MAPK, P38/MAPK, and ERK/MAPK pathways, reducing the secretion of inflammatory cytokines in mice with septic shock (57). Among mice in septic shock, Tang et al. found that calmodulin, the molecular chaperone of the Transient Receptor Potential Canonical (TRPC) channels, uncouples in the absence of Trpc1 or Trpc6 and binds TLR4, blocking the JNK/MAPK, P38/MAPK, ERK1/2/MAPK, and NF-κB pathways, thereby inhibiting the release of downstream inflammatory cytokines. This suggests that targeting TRPC using small-molecule inhibitors, such as SKF96365, could be a novel therapeutic strategy to suppress inflammation (58).

However, Shin et al. found that p38 MAPK negatively regulates the NLRP3 inflammasome by controlling Ca2+ mobilization; inhibition of P38/MAPK promotes activation of the NLRP3 inflammasome and enhances the activation of caspase 1 and the production of IL-1β and IL-18 in mice with sepsis. This leads to increased cytoplasmic Ca2+ and excessive uptake of mitochondrial Ca2+, resulting in increased mitochondrial damage, which is associated with excessive activation of the NLRP3 inflammasome (59). Hence, whether inhibition of the toll-like receptor family can be used as an anti-inflammatory approach requires further investigation.

The NOD-like receptor family plays a role in inflammation in septic shock. Wei et al. discovered that nitisinone (NTBC) induced the accumulation of the Phe/Tyr catabolic intermediate 4-hydroxyphenylpyruvate (4-HPP), thereby inhibiting NLRP3 activation and release. This, in turn, inhibited IL-1β release to suppress inflammation and effectively reduced septic shock induced by LPS in mice (60). This study suggests that inhibition of NLRP3 may be effective as a targeted anti-inflammatory therapy. In their study, Wang et al. found that silencing the NLRC4 gene using NLRC4-siRNA inhibited the activation of the nodular receptor (NLR) pathway and reduced levels of IL-1β, TNF-α, and IL-6, thereby reducing the septic shock-induced inflammatory response in mice (61).

IKKβ is involved in the assembly of IKK kinase, which plays an important part of the NF-κB signaling pathway. Chen et al. found that ellipticine (ELL) inhibited IKKβ and thus the NF-κB pathway; this inhibition of IKKβ also induced cellular autophagy to counteract inflammation and improve survival in mice with septic shock (62). Elkamhawy et al. designed and synthesized a thiazolidine-2,4-dione-type irreversible metamorphic IKKβ inhibitor that reduced mortality in septic shock mice, and this was more effective than reversible inhibitors, highlighting the potential of synthetic drugs in targeted therapy (63). The efficacy and safety of these IKKβ inhibitors, however, still need further validation.

Urbahn et al. found that TNF-α and IL-6 were reduced in the plasma of septic shock mice knocked out of the PLD1 gene, and mortality was also lesser (64). Hwang et al. developed A3373, a potent inhibitor of PLD1, and this inhibitor was found to selectively bind to PLD1 to inhibit PLD1 activity (65). In our opinion, this inhibitor may also be effective in treating septic shock.

FH15 belongs to the fatty acid-binding protein (FABP) of schistosomiasis and is a recombinant protein with a mass of 14.5 kDa. In a septic shock model in mice, Marcos et al. found that FH15 reduced serum cytokines and chemokine storms, with serum levels of cytokines including IL-1β, IL-3, IL-6, IL-12p70, TNF-α, IFN-γ, and serum levels of chemokines including MCP-1, MIP-1, and KC decreasing. These results suggest that FH15 is a candidate drug to counteract inflammation in septic shock by decreasing serum cytokines (66).

There are other types of potential targets for the targeted treatment of septic shock inflammation. Baranowsky et al. found that procalcitonin promotes inflammation by causing γδ T cells to secrete IL-17A through the calcitonin gene-related peptide (CGRP) receptor, and using olcegepant to antagonize the CGRP receptor improves survival in mice with septic shock (67). However, Messerer et al. found that the use of osigepan in a septic shock model in pigs reduced survival in this model because of the antagonistic effect of osigepan on the CGRP receptor; however, while reducing inflammation, it caused damage to the cardiovascular system (68). It may be possible to mitigate the damage caused by olcegepant to the cardiovascular system if engineered EVs are used to target γδ T cells for olcegepant transport.

In their study, Hara et al. found that mice knocked out of the Mint3 gene had defective macrophage function, as evidenced by reduced hyperactivation of macrophages in the presence of inflammation, and that knockout of the Mint3 gene resulted in increased survival in septic shock mouse models. The action of the Mint3 protein activates HIF-1 by binding to FIH-1, and the effect of Mint3 knockout on macrophages may be due to reduced macrophage ATP production as a result of inhibition of HIF-1 (69). Sakamoto et al. identified naphthofluorescein, an inhibitor that binds to the Mint3 protein and inhibits the production of Mint3-dependent inflammatory cytokines in macrophages of septic-shocked mice, suggesting that the Mint3 protein is a potential target (70).

In **Table 2**, we have summarized these cargos mentioned in the above section that can be loaded into engineered EVs to alleviate inflammation in septic shock.

**Table 2:** Cargos loaded in engineered EVs may alleviate inflammation in septic shock.

Cargo	Target	Models	Note	Reference
PDK2-IN	PDK2	mice	Inhibiting PDK2 reduces the secretion of inflammatory cytokines in mice with septic shock.	(57)
SKF96365	TRPC channels	mice	Inhibiting TRPC channels can decrease the secretion of inflammatory cytokines in septic shock mice.	(58)
nitisinone	NLRP3	mice	Inhibiting NLRP3 activation and release can suppress the release of IL-1β in mice with septic shock.	(60)
NLRC4-siRNA	NLRC4	mice	Silencing the NLRC4 gene can reduce septic shock-induced inflammatory response in mice by decreasing levels of IL-1β, TNF-α, and IL-6.	(61)
ellipticine, thiazolidine-2,4-dione-type irreversible metamorphic	IKKβ	mice	Inhibiting IKKβ can reduce inflammatory responses in septic shock mice by decreasing cellular autophagy and improving the survival rate of septic shock mice.	(62, 63)
A3373	PLD1	mice	Inhibiting PLD1 can decrease TNF-α and IL-6 levels in the plasma of septic shock mice, thereby reducing the mortality rate in septic shock mice.	(64, 65)
FH15	NA	mice	FH15 can reduce serum cytokine and chemokine storms in septic shock mice.	(66)
olcegepant	CGRP receptor	mice	Inhibiting CGRP receptors can reduce the secretion of IL-17A by γδT cells in septic shock mice, leading to an improvement in the survival rate of septic shock mice.	(67)
naphthofluorescein	Mint3	mice	Inhibiting Mint3 can decrease the generation of inflammatory cytokines in septic shock mice, leading to an improvement in the survival rate of septic shock mice.	(69, 70)

NA, not appliable.

## The role of cargos loaded into engineered EVs in suppressing organ damage in septic shock

Multi-organ damage, blood hypercoagulation, immunosuppression, and endothelial barrier dysfunction occur frequently in septic shock (56). A number of possible therapeutic targets have been identified for these conditions.

Neeli et al. highlighted the role of peptidylarginine deaminase (PAD) in catalyzing the formation of citrullinated histone H3 (CitH3) from histone H3, which is involved in neutrophil extracellular trap (NET) formation (71). In their study, Zhao et al. found that Cl-amidine, a PAD inhibitor, significantly reduced bone marrow and thymus atrophy, promoted bone marrow intrinsic immune cell recovery, increased blood/liver bacterial and blood monocyte clearance, reduced pro-inflammatory cytokine production, exerted protective effects, and increased survival among mice in septic shock (72).

Z-dependent protease inhibitor (ZPI) is a type of anticoagulant protein targeted to reduce coagulation factors Xa and Xia, and its plasma concentration rises in the inflammatory setting as an acute phase reactant. Bianchini et al. found that although plasma ZPI concentrations were higher in patients in septic shock compared to normal subjects, the ZPI present in the plasma of patients with severe sepsis was hydrolyzed by neutrophil elastase (NE) and purified NE on the surface of neutrophil extracellular traps (NETs). This resulted in the cleavage and inactivation of plasma ZPI, and hence, plasma ZPI concentrations were lower in patients with severe sepsis, and the blood of these patients was prone to clotting (73).

**Figure 3:**
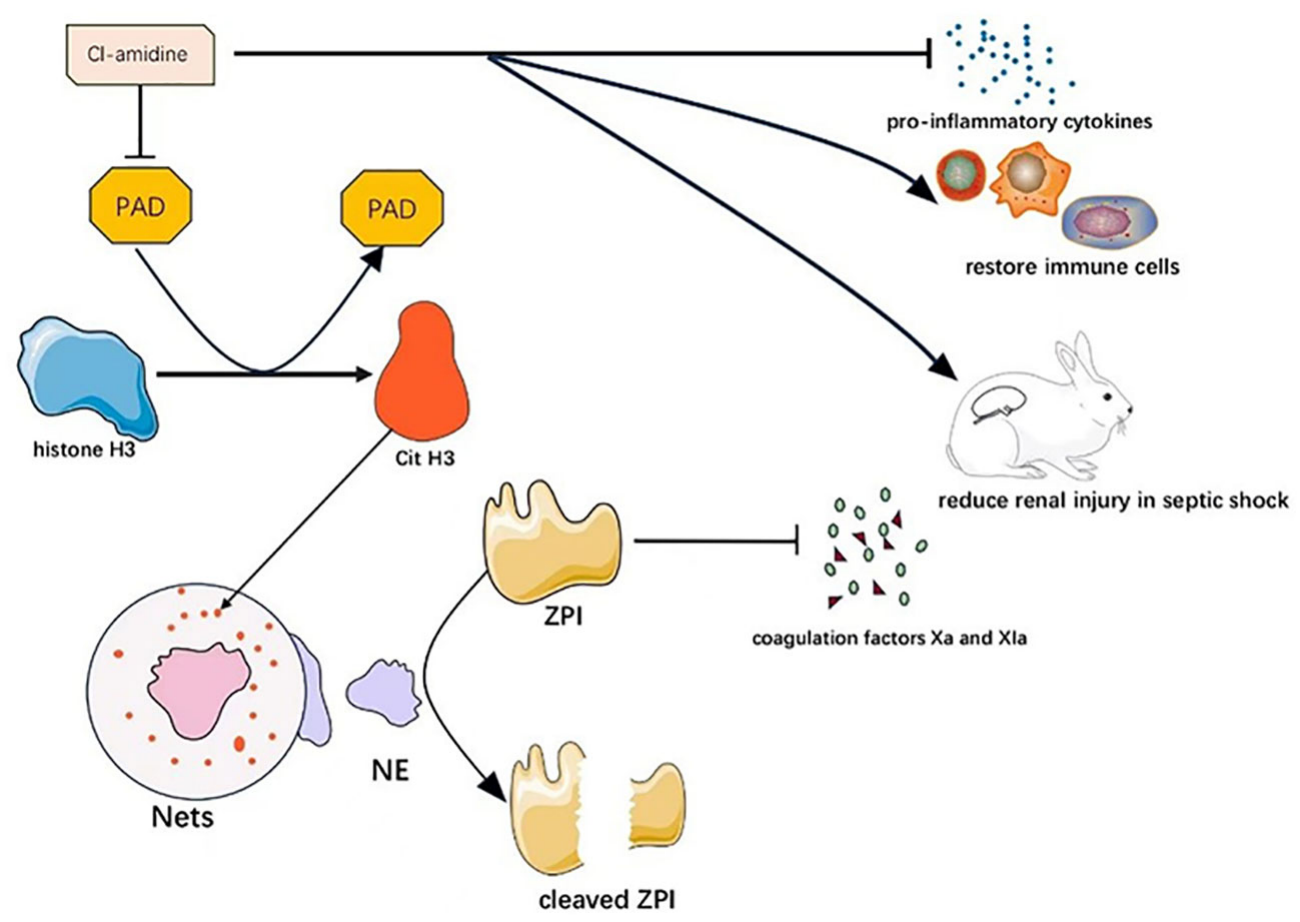
Mechanism of PAD as a potential therapeutic target in septic shock. Cl-amidine can alleviate septic shock through multiple mechanisms by inhibiting PAD.

It has been hypothesized that NETs contain a type of active elastase that produces plasminogen fragments with inhibitory activity that prevent the binding of fibrinogen to fibrin and reduce fibrinogen concentration and fibrinolysis formation. This deficiency in fibrinolysis may contribute to the impaired microthrombolysis in septic shock (74). Therefore, we hypothesize that Cl-amidine may also reduce the risk of clot formation in septic shock patients by reducing the production of NETs, which may have anticoagulant effects. Therefore, we believe that PAD is a highly attractive therapeutic target. The mechanism of PAD as a potential therapeutic target in septic shock is shown in **Figure 3**.

The NOD-like receptor family also plays a role in organ damage. Silencing of the NLRC4 gene mediated by the use of NLRC4-siRNA inhibited activation of the NOD-like receptor (NLR) pathway to reduce septic shock-induced lung injury. This inhibition reduced dendritic cell maturation and entry into the cell cycle, increased dendritic cell death (apoptosis), and reduced dendritic cell-mediated immune responses (61).

Zhang et al. found that mitochondrial homeostasis was maintained by aldehyde dehydrogenase (ALDH2). This maintenance was achieved through aldehyde scavenging and antioxidant effects of ALDH2, and ALDH2 also inhibited NLRP3 inflammatory vesicle activation and caspase-1/gsdmd-dependent septic shock-induced cardiac myocyte pyroptosis. The authors suggest that ALDH2 could inhibit the mitochondrial-inflammasome pathway through the clearance of aldehydes and that ALDH2 blocks the nuclear translocation of histone deacetylase 3 (HDAC3) into the mitochondria and the subsequent deacetylation of HADHA, thereby protecting against myocardial pyroptosis induced by septic shock (75).

As a new and excellent donor of hydrogen sulfide (H2S), GYY4137 [GYY, morpholin-4-4-methoxyphenyl (morpholino) phosphinodithioate] can deliver hydrogen sulfide *in vivo*, and Li et al. discovered that GYY attenuates inflammation and acute lung injury in septic shock through inhibition of the PDGFRβ/Akt/NF-κB/NLRP3 pathway (76). The results of these two studies indicate that NLRP3 can be a potential target for reducing organ damage in septic shock.

In a septic shock model in canines, Wang et al. discovered that elevated cell-free hemoglobin (CFH) alone increased mortality because CFH exacerbated pneumonia by increasing the utilization of released iron by infected bacteria, and CFH also scavenged nitric oxide (NO), causing pulmonary hypertension and cardiogenic shock. These effects of CFH exacerbate shock and multi-organ failure and increase septic lethality, but there are no specific inhibitors of CFH (77).

Zhang et al. found that resveratrol improved vasodilatory responsiveness and protected tissue perfusion and vital organ function through upregulation of endothelial-type nitric oxide synthase (eNOS) expression and downregulation of inducible nitric oxide synthase (iNOS), rac-1, and HIF-1α. They demonstrated that eNOS expression was increased by inhibition of rac-1 and HIF-1α (78). This study demonstrates the possible facilitative role of eNOS in increasing vasodilatory responsiveness in patients in septic shock.

In their study, Wang et al. discovered that the histone demethylase KDM1A/LSD1 suppressed inflammation by negatively regulating the response of hematopoietic stem cells to inflammation during septic shock in mice. Another result was that inflammation-induced deletion of lysine-specific demethylase-1 (LSD1) leads to a septic shock phenotype as well as acute death, a deletion caused primarily by inflammation-induced microRNAs. They found that blocking inflammation-inducing miRNAs using antisense miRNAs of inflammation-inducing miRNAs increased LSD1 levels and reduced death in septic shock mice, indicating the potential of LSD1 activators and inhibitors of inflammation-inducing miRNAs in treating septic shock (79).

Triggering receptor expressed on myeloid cells 2 (TREM2) inhibited ACTH-induced macrophage-secreted EV-mediated steroidogenesis in adrenocortical cells during early septic shock, as found by Ye et al. They also found that tissue perfusion in TREM2-deficient mice during acute septic shock was improved by enhanced corticosterone biosynthesis, but mortality in TREM2-deficient septic shock mice was not statistically different from that in wild-type mice, meriting further study to determine whether TREM2 could be a potential target of septic shock (80).

## Strengths and limitation

This paper presents a clear point of view, that is, EVs are potential targets for septic shock and many other diseases. Pioneers and recent studies are used in the manuscript, and the results of each study are explained accordingly. However, there are some limitations to this paper: First, many of the references applied in this paper are published in lower-ranked journals, which may somewhat limit the support for the concept. In addition, the manuscript also fails to show experimental evidence of the existence of EVs and of their relationships and/or correlations with sepsis and other diseases, damaged cells encountered in those diseases, as well as intact cells of healthy individuals. This is what we need to study extensively and comprehensively in the future.

## Conclusion and future prospects

### Conclusion

The perception of extracellular vesicles (EVs) has transformed significantly since their initial discovery, evolving from being viewed solely as carriers of cellular metabolic waste to now being considered essential mediators of intercellular communication and playing a vital role in both physiological and pathological processes. Research into EVs has advanced rapidly in recent years, and researchers have made significant discoveries in investigating the role that EVs may play in disease development, diagnosis, and treatment. We have provided a comprehensive delineation of the dual functionality exhibited by EVs within the context of septic shock. Our discussion encompasses the prospective utility of EVs as diagnostic markers for septic shock, the plausible application of EVs in targeted therapeutic interventions for septic shock, and a summary of potential cargoes amenable to loading into engineered EVs.

### Future prospects

EVs from different sources play various roles in the pathophysiology of septic shock, including inflammatory response, immune response, oxidative stress, and cellular communication, among others. However, the effect of the EV and its cargo on the severity of septic shock, the specific timing of its role in septic shock, and whether its role will change with the disease progression remain unclear. Moreover, research on EVs often focuses on specific types or a limited number of EVs, while there are multitudinous types of EVS. Therefore, this diversity necessitates extensive and comprehensive studies to further explore the detailed mechanisms by which EVs contribute to septic shock. In our opinion, EVs will also be pivotal in the future for disease diagnosis and treatment.

Future research on EVs as diagnostic markers should focus on three categories of diseases: diseases requiring early screening, diseases requiring monitoring of disease progression, and diseases requiring prognostic judgment. Septic shock is a disease involving the latter two scenarios. The potential applications of engineered EVs in treating septic shock are undoubtedly broader than those of native EVs, but the challenge of clinical manufacturing of engineered EVs persists, and this is the main issue limiting the use of engineered EVs in clinical therapy. We believe this problem can be solved, as there have been many studies in recent years on the large-scale production of clinical-grade exosomes based on bioreactors compliant with Good Manufacturing Practice (GMP) standards. Some of the studies we have reviewed have achieved significant results in animal experiments. Given the complicated pathogenesis of septic shock, we anticipate that advancements in large-scale production techniques aligned with GMP standards may indeed pave the way for the clinical translation of engineered EV therapies, offering new avenues for more effective and targeted treatments for septic shock in the future.

## Author contributions

MC: Conceptualization, Writing – original draft, Writing – review & editing. MS: Data curation, Formal analysis, Writing – original draft. BZ: Formal analysis, Writing – original draft. HJ: Conceptualization, Writing – review & editing.
